# *LRRK2* N551K and R1398H variants are protective in Malays and Chinese in Malaysia: A case–control association study for Parkinson's disease

**DOI:** 10.1002/mgg3.604

**Published:** 2019-09-05

**Authors:** Aroma Agape Gopalai, Jia Lun Lim, Hui‐Hua Li, Yi Zhao, Thien Thien Lim, Gaik B. Eow, Santhi Puvanarajah, Shanthi Viswanathan, Mohamed Ibrahim Norlinah, Zariah Abdul Aziz, Soo Kun Lim, Chong Tin Tan, Ai Huey Tan, Shen‐Yang Lim, Eng‐King Tan, Azlina Ahmad Annuar

**Affiliations:** ^1^ Faculty of Medicine, Department of Biomedical Science University of Malaya Kuala Lumpur Malaysia; ^2^ Health Services Research Singapore General Hospital Singapore Singapore; ^3^ Centre for Quantitative Medicine Duke‐NUS Medical School Singapore Singapore; ^4^ Department of Clinical Translational Research Singapore General Hospital Singapore Singapore; ^5^ Island Hospital George Town Malaysia; ^6^ Department of Neurology Hospital Pulau Pinang Penang Malaysia; ^7^ Department of Neurology Hospital Kuala Lumpur Kuala Lumpur Malaysia; ^8^ Hospital University Kebangsaan Malaysia Kuala Lumpur Malaysia; ^9^ Department of Medicine Hospital Sultanah Nur Zahirah Kuala Terengganu Malaysia; ^10^ Faculty of Medicine, Department of Medicine University of Malaya Kuala Lumpur Malaysia; ^11^ Faculty of Medicine, Division of Neurology and the Mah Pooi Soo & Tan Chin Nam Centre for Parkinson's & Related Disorders University of Malaya Kuala Lumpur Malaysia; ^12^ Department of Neurology Singapore General Hospital Singapore Singapore; ^13^ National Neuroscience Institute and Duke‐NUS Graduate Medical School Singapore Singapore

**Keywords:** LRRK2, N551K, Parkinson's disease, R1398H

## Abstract

**Background:**

The* LRRK2* gene is associated with Parkinson's disease (PD) as a number of mutations within the gene have been shown to be susceptibility factors. Studies on various global populations have determined that mutations such as G2019S, G2385R, and R1628P in *LRRK2* increase the risk of developing PD while the N551K‐R1398H haplotype is associated with conferring protection against developing PD. Here we report a study looking at the N551K and R1398H variants for the first time in the Malaysian population.

**Methods:**

Cases (523) which conformed to the United Kingdom PD Brain Bank Criteria for PD were recruited through trained neurologists and age‐ and ethnically matched controls (491) were individuals free of any neurological disorder. The N551K and R1398H mutations were genotyped using the Taqman SNP genotyping assay.

**Results:**

A significant protective association for N551K was found in those of Malay ancestry, with a protective trend seen for R1398H. A meta‐analysis of Chinese individuals in this cohort with other published cohorts of Chinese ancestry indicated a significant protective role for N551K and R1398H.

**Conclusion:**

This study reports that the N551K‐R1398H haplotype is also relevant to the Malaysian population, with a significant protective effect found in those of Malay and Chinese ancestries.

## 1 INTRODUCTION

Parkinson's disease (PD) is an age‐related neurodegenerative disease, caused by the loss of dopaminergic neurons in the substantia nigra pars compacta of the brain. The loss of the dopaminergic neurons leads to a range of movement problems including rigidity, bradykinesia, and tremors.

The leucine‐rich repeat kinase 2 (*LRRK2)* gene has been extensively studied in relation to both familial and sporadic forms of Parkinson's disease. The PD mutation database reports 127 mutations in this gene, with the G2019S mutation accounting for 40% of North African Arab and 20% of Ashkenazi Jewish PD cases (Ozelius et al., 2006). The G2019s mutation has been consistently shown to result in hyperactivation of the LRRK2 kinase, which has been associated with defects in protein synthesis and degradation, apoptosis, inflammatory responses, and oxidative damage (Rui et al., 2018; Smith et al., 2006). However, this mutation is almost completely absent in Asian PD populations studied thus far (Japanese, Chinese, and Koreans) (Bekris, Mata, & Zabetian, 2010; Guedes et al., 2010; Lesage et al., 2006). In contrast, the G2385R and R1628P mutations are relatively common in Asian PD patients, suggesting differing ethnic‐specific patterns of inheritance for *LRRK2* mutations (Gopalai et al., 2014; Zhang et al., 2017).

The N551K and R1398H variants were first described in a PD study looking at linkage disequilibrium within *LRRK2 *(Paisan‐Ruiz et al., 2006). A multicenter case–control study suggested that individuals carrying the N551K (c.1653C > G, rs7308720) and R1398H (c.4193G > A, rs7133914) variants had a 20% reduced risk of developing PD (Ross et al., 2011). This was replicated in two Asian studies in Singapore and Taiwan (Tan et al.., 2010; Wu et al., 2013). These variants have not previously been screened in a Malaysian PD population and constitutes a significant gap in our understanding of the associated genetic factors in this population. With the advent of targeted therapies (Chan & Tan, 2017), an improved understanding of the genetic and mechanistic factors underlying PD is becoming increasingly important. Therefore, this study aimed to investigate the association between these protective alleles with the risk of PD in a multi‐ethnic Malaysian population.

**Figure 1:**
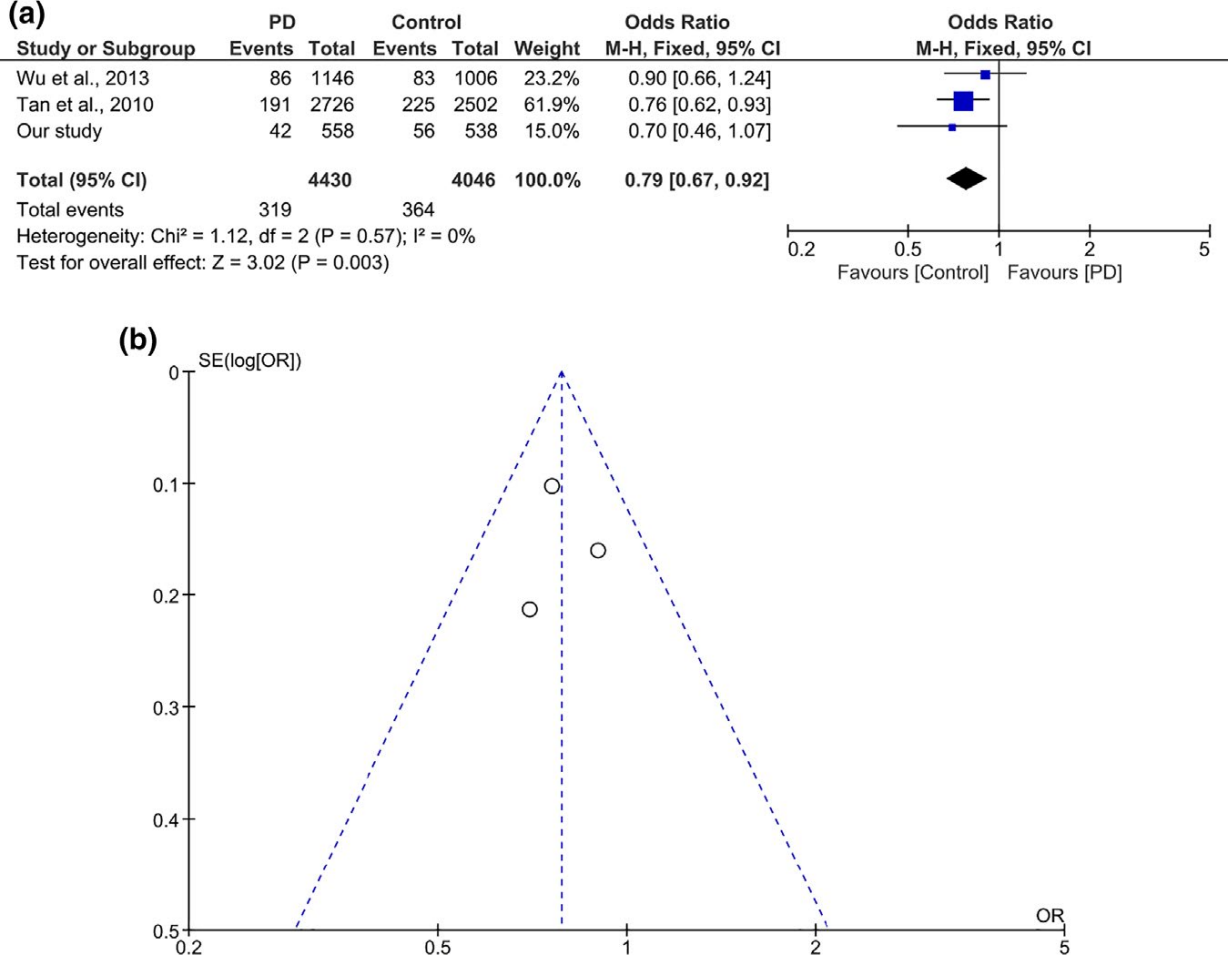
(a) Forest plot of pooled analysis for N551K among Chinese PD cohort. (b) Forest plot of pooled analysis for R1398H among Chinese PD cohort

**Figure 2:**
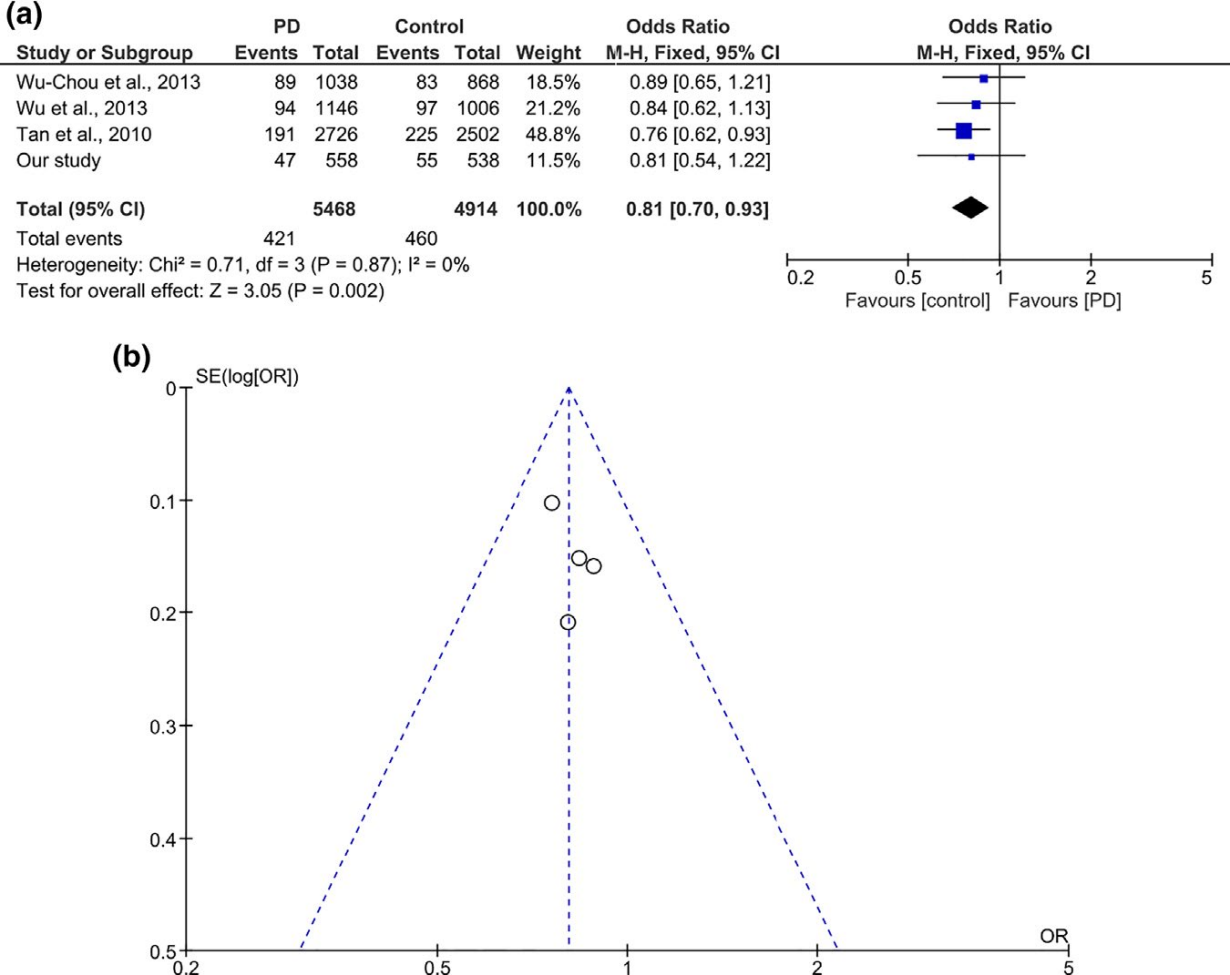
(a) Funnel plot of pooled analysis using fixed effect model for N551K among Chinese PD cohort. (b) Funnel plot of pooled analysis using fixed effect model for R1398H among Chinese PD cohort

## 2 MATERIALS AND METHODS

### 2.1 Sample recruitment and genetic analysis

A total of 523 PD cases and 491 controls were screened. The patients were recruited from neurology clinics throughout Peninsular Malaysia. PD patients were diagnosed based on the United Kingdom PD Brain Bank Criteria by movement disorder specialists or neurologists with an interest in PD. Control subjects were recruited from spouses and from outpatient clinics. The controls were age and gender‐matched and were not suffering from any neurological disorder. Institutional ethical approval was obtained and all participants provided written informed consent.

The N551K and R1398H variants were genotyped using TaqMan® SNP genotyping assays (Applied Biosystems) on a 7,500 Fast Real‐Time PCR machine. Genotypes were confirmed by sequencing in a subset of 20 individuals to confirm the genotypes and determine the error rate.

Statistical analysis was performed using open‐source software (OpenEpi) while Review Manager 5 (RevMan 5) (Collaboration, 2014) was used to conduct the meta‐analysis among the Chinese cohort. Heterogeneity among the studies was assessed with the *I^2^* statistics (Higgins & Thompson, 2002).

## 3 RESULTS AND DISCUSSION

The mean age at PD diagnosis was 57.4 ± 11.8 years and the mean age of controls was 59.3 ± 9.4 years (*p* = 0.0048). Sixty per cent of PD patients and 51% of controls were male. The cohort consisted of 168 Malay PD cases, 133 Malay controls, 279 Chinese PD cases, 269 Chinese controls, 76 Indian PD cases, and 89 Indian controls.

Linkage analysis (Haploview 4.2) indicated that N551K and R1398H are in linkage disequilibrium (D’ = 0.959, *r* ^2^ = 0.906), similar to what has been reported by Tan et al., 2010 (Tan et al., 2010). Genotypes for both variants were in Hardy–Weinberg equilibrium. The error rate of the assay was 0%.

The mutant alleles for both variants were more frequent in the controls by almost twofold. An odds ratio (OR) of 0.623 (95% CI 0.44–0.88, *p* = 0.007) was obtained for N551K, and an OR of 0.699 (95% CI 0.50–0.98, *p* = 0.036) was obtained for R1398H, suggesting a reduced risk of developing PD in carriers (Table 1).

**Table 1:** Details of genotypes and allele frequencies of N551K and R1398H by ethnicity

	PD cases			Controls		
	Malay	Chinese	Indian	Malay	Chinese	Indian
N551K (c.1653C>G), rs7308720						
Wild type (C/C)	155	239	72	113	214	81
Heterozygous mutant (C/G)	13	38	4	18	54	8
Homozygous mutant (G/G)	–	2	–	2	1	–
Allelic frequency (%)						
Wild type (C)	96.1	92.5	97.4	91.7	89.6	95.5
Mutant (G)	3.9	7.5	2.6	8.3	10.4	4.5
R1398H (c. 4193G>A), rs7133914						
Wild type (G/G)	155	233	71	115	215	80
Heterozygous mutant (G/A)	13	45	5	16	53	8
Homozygous mutant (A/A)	–	1	–	2	1	1
Allelic frequency (%)						
Wild type(G)	95.6	91.5	96.7	92.5	89.8	93.6
Mutant (A)	4.4	8.6	3.3	7.5	10.2	5.4

When analyzed according to ethnicities, a protective association for N551K was detected in Malays (OR 0.446, 95% CI 0.22–0.90, *p* = 0.025). No significant difference was found between the Chinese case and controls (OR 0.700, 95% CI 0.46–1.07, *p* = 0.096). The Indian subgroup showed a similar, albeit nonsignificant trend (OR 0.574, 95% CI 0.17–1.95, *p* = 0.373), likely due to the small sample size. When we performed a meta‐analysis for N551K on the combined Chinese datasets from this study, and those by Tan et al. and Wu et al., the analysis showed a significant protective effect with an OR of 0.79 (95% CI 0.67‐0.92, p = 0.003 (Table 2, Figures 1a, 2a). No heterogeneity was detected amongst the studies included in the meta‐analysis for N551K (p_heterogeneity_ = 0.57, *I^2^* = 0%). Meta‐analysis on Malay and Indian samples was not performed as there are no other published studies on these populations for N551K.

**Table 2:** Summary of published reports on genetic studies of N551K and R1398H in Parkinson's disease and meta‐analysis results

Study	Country/Population	Sample size	Results
**N551K** (c.1653C>G), rs7308720			
Tan et al., 2010 (Han Chinese)	Singapore	250 PD, 250 controls	OR 0.60 (*p* = 0.019)
	Singapore	192 PD, 192 controls	OR 0.92 (*p* = 0.757)
	Taiwan	293 PD, 299 controls	OR 0.62 (*p* = 0.021)
	China	628 PD, 510 controls	OR 0.91 (*p* = 0.570)
	Combined	1363 PD, 1251 controls	OR 0.74 (*p* = 0.004)
Ross et al., 2011	Caucasian	6995 PD, 5595 controls	OR 0.88 (*p* = 0.025)
	Asian	1376 PD, 962 controls	OR 0.73 (*p* = 0.0017)
	Arab‐Berber	240 PD, 372 controls	OR 0.83 (*p* = 0.47)
Wu et al., 2013	Taiwan	573 PD, 503 controls	OR 0.91 (*p* = 0.577)
Current study	Malaysia	523 PD, 491 controls	OR 0.623 (*p* = 0.007)
Meta‐analysis on Chinese samples	This study,	2215 PD, 2023 controls	OR 0.79 (*p* = 0.003)
	Tan et al., and		
	Wu et al		
**R1398H** (c. 4193G>A), rs7133914			
Tan et al., 2010 Han Chinese	Singapore	250 PD, 250 controls	OR 0.64 (*p* = 0.038)
	Singapore	192 PD, 192 controls	OR 0.85 (*p* = 0.559)
	Taiwan	293 PD, 299 controls	OR 0.64 (*p* = 0.033)
	China	628 PD, 510 controls	OR 0.90 (*p* = 0.566)
	Combined	1363 PD, 1251 controls	OR 0.75 (*p* = 0.005)
Ross et al., 2011	Caucasian	6995 PD, 5595 controls	OR 0.89 (*p* = 0.034)
	Asian	1376 PD, 962 controls	OR 0.73 (*p* = 0.002)
	Arab‐Berber	240 PD, 372 controls	OR 1.00 (*p* = 1.00)
Wu et al., 2013	Taiwan	573 PD, 503 controls	OR 0.84 (*p* = 0.2418)
Wu‐Chou et al., 2013	Taiwan	519 PD, 434 controls	OR 0.89 (*p* = 0.45)
Current study	Malaysia	523 PD, 491 controls	OR 0.699 (*p* = 0.036)
Meta‐analysis on Chinese samples	This study,	2734 PD, 2457 controls	OR 0.81 (*p* = 0.002)
	Tan et al.,		
	Wu et al., and		
	Wu‐Chou et al.		

When analyzing R1398H according to ethnicities, the protective association was detectable with borderline significance in Malays (OR 0.495, 95% CI 0.24–1.01, *p* = 0.055), but the results were not significant in the Chinese (OR 0.808, 95% CI 0.54–1.22 *p* = 0.306) and Indians (OR 0.571, 95% CI 0.19–1.71 *p* = 0.317). We performed a meta‐analysis of R1398H (combined Chinese datasets from this study, and those by Tan et al., Wu et al., and Wu‐Chou et al.) and this showed an OR of 0.81 (95% CI 0.70‐0.93, p = 0.002), (Table 2, Figures 1b, 2b). Similar to N551K, meta‐analysis on Malays and Indians was not possible as this is the first report on these populations for these variants. No heterogeneity was detected amongst the studies included in the meta‐analysis for R1398H (p_heterogeneity_ = 0.87, *I^2^* = 0%).

## 4 CONCLUSION

We have previously reported an association between PD in our Malaysian cohort with the risk alleles G2385R and R1628P within the *LRRK2* gene (Gopalai et al., 2014). However, the association with the R1398H and N551K protective alleles have not previously been determined in this population. Here we report that N551K variant is associated in a protective manner in the Malay population, with the R1398H variant also showing a similar protective trend. N551K and R1398H showed a significant protective association when we pooled our Chinese cohort with other reported studies. The N551K‐R1398H association was unable to be detected in the Malaysian Indian cohort, likely due to the relatively small sample size.

The exact mechanism(s) underlying the protective effects of the N551K‐R1398H haplotype are not clear at present. The N551K variant is not within any domain of the LRRK2 protein, but is in linkage disequilibrium with the R1398H variant, which lies within the Ras‐of‐complex (ROC) GTPase domain, and enables the binding of guanine nucleotides via a phosphate‐binding motif.

The kinase activity of LRRK2 is modulated by GTPase activity, GTP hydrolysis, and GTP binding (Cookson, 2010). Pathogenic mutations in *LRRK2* such as G2019S elevate the level of kinase activity (West et al., 2005), which in turn has been shown to cause neuronal toxicity (Smith et al., 2006). Lower levels of GTPase activity lead to a lower level of LRRK2 kinase activity (Biosa et al., 2012). Studies on R1398H have indicated that it plays a role in decreasing GTP‐bound LRRK2, in addition to positive effects on axon outgrowth and activation of associated Wnt signaling pathways (Nixon‐Abell et al., 2016). This may be one mechanism through which it may be conferring a protective effect on the cell.

Apart from having a protective effect in PD, another study investigated a possible link with rapid eye movement‐sleep behavior disorder (RBD), a condition now regarded as a prodromal symptom of synucleinopathies, most commonly PD. In a case–control study involving 350 RBD patients and 869 controls, the N551K‐R1398H haplotype was significantly associated with a reduced risk of developing RBD (Bencheikh et al., 2018). No association was found in studies of Alzheimer's disease patients (Ng, Ng, Tan, Kandiah et al., 2018) or essential tremor (Ng, Ng, Tan, Prakash et al., 2018).

In conclusion, we show that consistent with other published reports on the protective effect of N551K and R1398H, these variants are also protective in the Malaysian Malay and Chinese ethnicities. Further studies will need to be done to determine the cellular mechanism of how this protective effect is mediated.

## CONFLICT OF INTEREST

The authors have no conflicts of interest to declare.
